# A sterile insect technique pilot trial on Captiva Island: defining mosquito population parameters for sterile male releases using mark–release–recapture

**DOI:** 10.1186/s13071-022-05512-3

**Published:** 2022-11-01

**Authors:** Danilo O. Carvalho, Rachel Morreale, Steven Stenhouse, Daniel A. Hahn, Maylen Gomez, Aaron Lloyd, David Hoel

**Affiliations:** 1 Insect Pest Control Subprogramme, Department of Nuclear Sciences and Applications, Joint FAO/IAEA Centre of Nuclear Techniques in Food and Agriculture, International Atomic Energy Agency, 1400 Vienna, Austria; 2 Lee County Mosquito Control District, 15191 Homestead Road, Lehigh Acres, FL 33971 USA; 3 Department of Entomology and Nematology, University of Florida, 1881 Natural Area Drive, Gainesville, FL 32611 USA

**Keywords:** Sterile insect technique, Mark–release–recapture, *Aedes aegypti*, Mosquito population size, Mosquito dispersal

## Abstract

**Background:**

The sterile insect technique (SIT), which involves area-wide inundative releases of sterile insects to suppress the reproduction of a target species, has proven to be an effective pest control method. The technique demands the continuous release of sterilized insects in quantities that ensure a high sterile male:wild male ratio for the suppression of the wild population over succeeding generations.

**Methods:**

For these releases, it is important to determine several ecological and biological population parameters, including the longevity of the released males in the field, the dispersal of the released males and the wild pest population size. The Lee County Mosquito Control District initiated a study in a 47-ha portion of Captiva Island (Florida, USA), an island with a total area of 230 ha, to define biological SIT parameters for *Aedes aegypti* (L.), an invasive disease-vectoring mosquito known to be difficult to control due to a combination of daytime biting activity, use of cryptic breeding habitats that are difficult to target with conventional night-time ultra-low volume methods, and emerging resistance to commonly used insecticides. Another goal was to assess patterns of dispersal and survival for laboratory-reared sterile *Ae. aegypti* males released over time in the pilot site. These parameters will be used to evaluate the efficacy of a SIT suppression program for *Ae. aegypti* on Captiva Island.

**Results:**

Over the course of seven mark-release-recapture studies using single- and multiple-point releases, 190,504 sterile marked males were released, for which the recapture rate was 1.5% over a mean period of 12 days. The mean distance traveled by sterile males of the local strain of *Ae. aegypti* that has colonized Captiva Island was 201.7 m from the release point, with an observed maximum traveled distance of 404.5 m. The released sterile mosquitoes had a probability of daily survival of 0.67 and an average life expectancy of ~ 2.46 days.

**Conclusions:**

These data together with the population size estimate and sterile:wild ratio provide a solid basis for planning the SIT operational phase which is aimed at mosquito population suppression.

**Graphical abstract:**

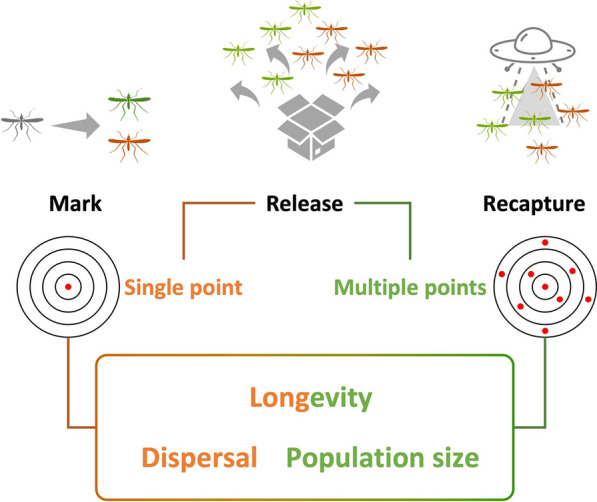

**Supplementary Information:**

The online version contains supplementary material available at 10.1186/s13071-022-05512-3.

## Background

The mosquito *Aedes aegypti* commonly occurs in and around homes and businesses in urban and suburban locations in many tropical and subtropical areas of the world. It is a major vector of viruses that can cause human disease, including dengue, chikungunya, Zika, and yellow fever viruses [1]. *Aedes aegypti* is of particular concern in Florida, USA, because of its role in the local transmission of Zika virus in 2016 in Miami, and limited local transmission of dengue in the state on several occasions in the last 20 years [2]. Even in areas where these arboviruses are rare or absent, *Ae. aegypti* is a serious nuisance because it lives in close proximity to people and exhibits aggressive biting behavior during lengthy periods of the day, which dampens enthusiasm for a broad range of outdoor activities [3].

A number of factors make *Ae. aegypti* difficult to control in urban and suburban environments. Breeding site removal and the effective application of larvicides can be difficult because *Ae. aegypti* females lay their eggs in many types of small cryptic oviposition sites that commonly occur in urban and suburban environments [4, 5]. Furthermore, *Ae. aegypti* adults tend to rest in protected, sheltered habitats that are not easily reached by ultra-low volume adulticides [6]. In addition to difficulties in reaching *Ae. aegypti* with traditional delivery methods for larvicides and adulticides, the prevalence of insecticide resistance due to the intensive use of insecticides has been increasingly documented in container-breeding mosquitoes worldwide, including *Ae. aegypti* in Florida [7, 8]. This decreasing efficacy of traditional mosquito control pesticides is also coupled with increasing public demand for the use of environmentally friendly, non-chemical pest control [9, 10].

Several innovative and targeted population suppression methods are under evaluation for use in integrative *Ae. aegypti* control, including the sterile insect technique (SIT). Although SIT has been used for many decades for several agricultural and livestock pests [11, 12], extensive efforts have recently been made to adapt the technique for mosquito control with the development of an International Atomic Energy Agency (IAEA) thematic plan along with a guidance framework for this subject devised by the World Health Organization and the IAEA [13]. Typically, SIT uses ionizing radiation to induce sterility in massive numbers of laboratory-reared male mosquitoes that, after being released in the field, compete with wild males to mate with wild females. With consistent releases of sterile males, SIT can reduce and even eventually dramatically suppress the wild population [14–16].

SIT requires an understanding of the size, spatial patterns of abundance, and dynamics of wild populations, as well as high-quality laboratory-reared sterile males for the successful suppression of mosquito populations [17]. Collecting baseline data on wild populations to characterize population densities in both high and low seasons is vital to estimating how many sterile males should be released to suppress the wild population each season. Indirect measurement of population density over space and time through entomological surveillance using traps to capture adult mosquitoes is the standard method for estimating mosquito population density [18, 19]. Fluctuations in mosquito populations are often tightly correlated with environmental conditions, such as rainfall patterns and temperature [20, 21]. However, documenting mosquito population dynamics alone is insufficient for successful SIT implementation because, ultimately, an understanding is required of how well laboratory-raised sterile males will perform in the field. Mark-release-recapture (MRR) studies are crucial for understanding wild male abundance, as well as for describing ecological parameters of released insects, such as the dispersal and survival dynamics of laboratory-raised sterile males in the field [21–24].

Lee County Mosquito Control District (LCMCD), located in Florida, USA, is responsible for controlling mosquito populations in an area of ~ 2033 km^2^ comprising ~ 98% of the county. Established in 1958 as an independent special taxing district, LCMCD operates a robust program aimed at mosquito suppression. Using an area-wide integrated pest management approach, efforts include chemical suppression of both the larval and adult stages, disease surveillance, mosquito population surveillance, and an insecticide-resistance monitoring program [25]. In 2017, LCMCD initiated a SIT pilot program in Captiva Island, Florida, USA, a ~ 230-ha barrier island, with the aim of establishing an operational SIT program for *Ae. aegypti* control. Following the phased conditional approach [18], LCMCD established a trapping network in 2017 for baseline data collection. Here we describe a series of MRR studies undertaken as preparatory steps for an operational suppression program. The MRR studies were performed using single-point releases [22, 26] to characterize sterile male dispersal patterns, and multiple-point releases [27, 28] were used to provide data on the longevity of released sterile males in the field, along with estimates of wild male population size on Captiva Island.

## Methods

### Colony rearing and irradiation

A colony of *Ae. aegypti* developed from field-collected eggs from Captiva Island and nearby Sanibel Island (381371.23 E, 2934234.89 N) was used for all the releases. The lab colony is refreshed annually after it reaches the 10th inbreeding generation by adding material from the field to reduce the side effects of colony inbreeding and to increase the chances of maintaining behavior similar to that of the wild population. A similar blood-feeding method previously described by our group was used for the production of eggs from adult females [29]. A nutrient broth of 200 mg Purina AquaMax Fry Powder in 500 ml water was used for egg hatching. After hatching, larvae were fed a slurry of Purina AquaMax Fry Powder (0.2, 0.3, 0.4, 0.6 and 0.6  mg/larva on days 2, 3, 4, 5 and 6, respectively). Upon pupation, the pupae were collected and separated by Fay-Morlan glass plate separators using pupal size differences to separate males from females [30]. Pupal weights were determined and used to estimate the number of mosquitoes for separate cohorts, which were divided into desired batch sizes for downstream treatment. Females were discarded. At least 30 h after pupation, male pupae were irradiated at a target dose of 52 Gy, which resulted in more than 99% sterility. Irradiation was carried out in a RS 2400 X-ray machine (Rad Source Technologies, Buford, GA), operated at 160 kV and 25 mA, with a dose rate of 13.89 Gy/min, following calibration procedures developed by IAEA [31]. Following sterilization, 2200 pupae were placed in emergence containers (2.37 L; height 14 cm, diameter 17 cm) with a 3-cm horizontal slit cut 1 cm from the bottom and containing 200 ml water. The smooth surface of the interior walls (with the exception of the lowest 2 cm) of the containers was lightly scuffed to provide a vertical area that was easier for mosquitoes to rest on. The lids were modified by replacing the inner 13.5-cm-diameter plastic center with 1-mm by 1-mm mesh screen. Once emergence was completed, the container was pressed just above the slit to create a small gap through which the water was gently poured out, and an absorbent piece of paper was inserted through the slit to soak up and remove all the remaining water. Pupal mortality was assessed from the material in the water. The adults were provided with 10% sucrose solution ad libitum via moist cotton placed on the screen portion of the lid until they were released. The insectary was maintained at a temperature of 27 ± 1 °C, 80% relative humidity, and 12-h light:12-h dark photoperiod.

### Study area

The MRR studies were carried out on Captiva Island between 2019 and 2021. Captiva is a ~ 306-ha barrier island off Florida’s southwest coast in Lee County (381371.23 E, 2934234.89 N, ~ 306 ha area). Captiva Island is connected to Sanibel Island to the south by an automotive bridge, and is bordered by the Gulf of Mexico to the west and Pine Island Sound to the east. Captiva has a subtropical climate with a rainy season in the warmest months that usually lasts from late May through September [average rainfall 19.91 cm, average temperature 27.46 °C (National Oceanic and Atmospheric Administration)]. The dry season, during which the temperatures are lower, stretches from October through April [average rainfall 5.44 cm, average temperature 20.3 °C (National Oceanic and Atmospheric Administration)]. There are 1108 housing units on Captiva, of which 188 are occupied and 920 vacant [32]. The island has a total human population of 318, but experiences high seasonal influxes of visitors from December through April [33]. A large resort area with a golf course, privately owned homes, condominiums, and restaurants occupies the north end of the island (~ 94 ha). The middle portion of Captiva Island has the highest density of residences, consisting of condominiums, townhomes and other types of privately owned homes of various sizes. In this area, there is also the greatest concentration of restaurants, many of which are at least partially open air, and other businesses. As Captiva is a narrow island, this area of high occupational density, which is situated in the widest section of the island, was considered the best site in which to perform the MRR studies. The buildings in the narrower southern part of the island (~ 210 ha) are primarily large privately owned homes.

### Mark-release-recapture

**Fig. 1:**
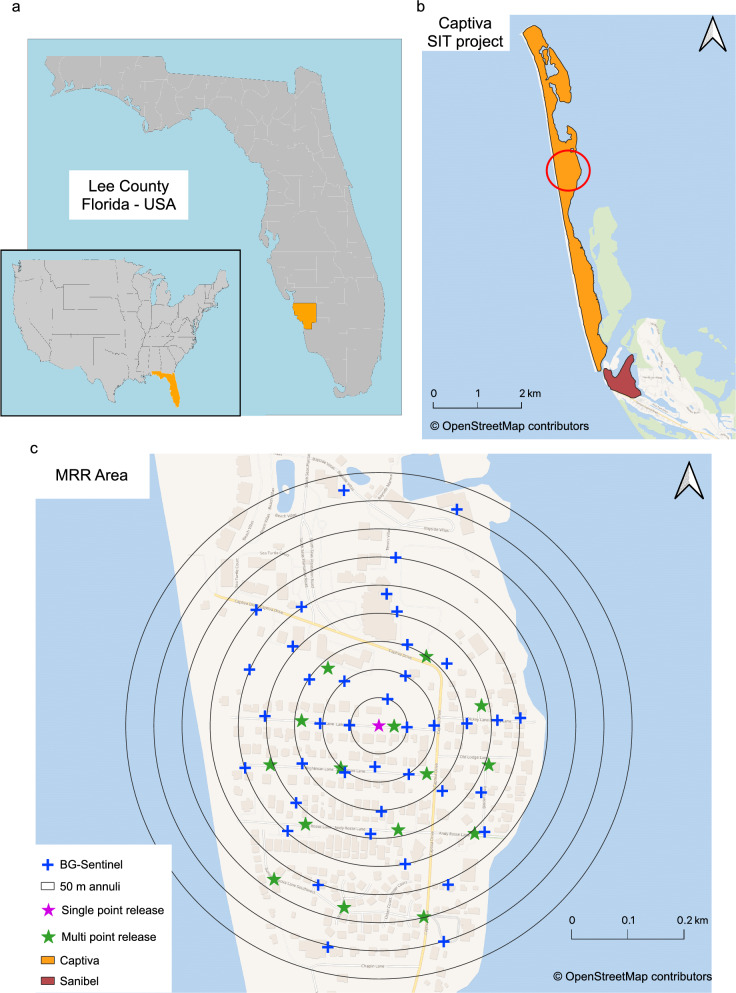
**a** Map showing the location of Lee County (in orange) in Florida. Inset shows the location of Florida (in orange) in the USA. **b** Captiva Island (in orange) and Sanibel Island (in maroon), Lee County. Approximate location of the ca. 47-ha mark–release–recapture (*MRR*) study site is circled in red. **c** Areas selected for MRR on Captiva Island were single-point release (pink star) and multiple-point release (green stars) positions. BG-Sentinel 2 trap (*BG-Sentinel*) positions are shown as blue crosses

The MRR studies were carried out across the year in an attempt to account for seasonal differences in the wild *Ae. aegypti* population. Based on the fluctuations observed in the baseline entomological data (in preparation), the estimated adult population density was categorized as generally low or high for each MRR event, with the additional descriptor of whether we expected the population to be entering a seasonal growth or decline phase. In total, between 2019 and 2021, seven MRR studies were performed at the widest part of the island (~ 47 ha; Fig. 1). Of these seven studies, three involved single-point releases (SP01, SP02, and SP03) and four multiple-point releases (MP01, MP02, MP03, and MP04). SP03 and MP04 correspond to one release because they were carried out on the same day; for these, differently colored fluorescent powder was used to mark the mosquitoes released from the center and those released from the surrounding points (Fig. 1c). Table 1 shows the differences between the MRR studies conducted on Captiva Island. These, minor, differences were due to facilitating and optimizing the studies according to the number of staff available and all the other activities that they needed to undertake.

**Table 1:** Summary of the mark–release–recapture (MRR) studies conducted at different time points on Captiva Island, Florida, USA

Release type	MRM Identifier	Date and time	Marking method	Release points (no.)	Containers	
					Total (no.)	Type
Single point	SP01	6 November 2019	Emergence in pots pre-marked with fluorescent powder	1	10	Emergence pot
		Morning				
	SP02	9 March 2020	Chilled males rolled in fluorescent powder		1	Screen cage
		Morning				
Multiple points	MP01	19 May 2020	Dusted with fluorescent powder	15	15	Emergence pot
		Morning				
	MP02	12 August 2020				
		Morning				
	MP03	12 April 2020				
		Afternoon				
Mixed	MP04	22 April 2021				
		Afternoon				
	SP03	22 April 2021		1	1	
		Afternoon				

*SP* Single-point release,* MP* multiple-point release

### Sterile mosquito marking

Three different approaches to marking sterile males were employed over the course of the study. Marking methods were modified and refined over time to ensure that males were marked sufficiently while minimizing an excess of fluorescent powder and handling of males.

#### Males marked through contact with fluorescent powder in conditioned pots

Approximately 0.25 g of fluorescent powder (Techno Glow, Ennis, TX) per emergence pot was manually applied in an even coat to the interior walls (excluding the lowest, unscuffed 2 cm) prior to the addition of sterile males. After applying the powder, 200 ml of water was carefully added to the containers to avoid splashing and contamination of the water by the powder. Post-irradiation, sterile male pupae were placed in the marked containers and allowed to eclose. The males marked themselves through contact with the fluorescent powder on the walls of the pots. Water was removed after eclosion was completed, as described previously. Males were provided with sucrose solution until the time of release, at ~ 48 h after eclosion.

#### Males marked through rolling in fluorescent powder

After eclosion, water was removed as described above, ~ 16 h prior to marking. On the day of release, emergence containers (containing 2200 males ~ 48 h after emergence) were placed in a refrigerator at ~ 1 °C for 1 h. After removal of the containers from the refrigerator, the males were transferred to similar containers with the interior surface pre-marked with ~ 0.25 g of fluorescent powder. Groups of sterile males were then gently rolled in these containers for 30 s to apply the marking dust. All 2200 males underwent this treatment prior to placement in a single screen cage (60 cm × 60 cm × 60 cm) for release. The males were provided with 10% sucrose solution until release.

#### Insufflation

Sterile males were released ~ 48 h after eclosion. On the day of release, the emergence containers (containing 2200 males) were placed in a refrigerator at ~ 1 °C for 7–10 min for brief immobilization of the males that allowed for their consistent marking. SP01 was the only release in which the males did not undergo this treatment. Once the containers had been removed from the refrigerator, adults hanging from the sides were gently dislodged by tapping. Sterile males were dusted with fluorescent powder (~ 1.6 g/15 containers) using a nasal aspirator which blew the powder inside the container to create a cloud that evenly settled on the immobilized mosquitoes, similarly to Balestrino et al. [34]. To ensure that large clusters of dye did not adhere to the males, the aspirator bulb was inserted into a fine mesh bag so that the powder was aerosolized. Once the pigment had been applied, the males in the containers were checked with ultraviolet light to confirm that they had been sufficiently marked, and the sucrose solution was replenished on the screened portion of the container lids until the males were released.

### Sterile marked mosquito releases

The number of release points depended on the type of release that was conducted, i.e. single-point releases at a single central point, and multi-point releases at 15 points (Fig. 1c). Releases SP01, SP02, MP01, and MP02 were conducted in the morning (0900–1100 hours), while releases SP03, MP03, and MP04 were performed in the afternoon (1200–1400 hours). For releases, lids were removed from the containers at the designated locations, and the mosquitoes were allowed to fly out. Gentle agitation was applied to encourage any remaining males to leave the container after the initial bout of dispersal. The container lids were replaced after the mosquitoes had been allowed to disperse from the container for 5–10 min. Any remaining mosquitoes, which included dead mosquitoes and mosquitoes that were alive but did not fly out of the containers after gentle agitation, were retained to determine mortality. All mortalities (pupal and adult) were recorded and subtracted from the initial estimated release number to improve the accuracy of subsequent calculations.

### Sterile marked mosquito recapture

All MRR events took place at the exact locations shown in Fig. 1 for trapping mosquitoes, for the duration of these studies. Forty BG-Sentinel 2 traps with lure (Biogents, Regensburg, Germany) were set outdoors in a radial pattern from a central location (Fig. 1c) with the traps arrayed in nine concentric annuli at 50-m intervals from the central point, with a maximum trap distance of 420 m [35, 36]. Trap servicing began 24 h after release and was carried out daily, with catch bags and batteries replaced approximately every 24 h. For all the studies, trap servicing was conducted until no marked mosquitoes were caught for two consecutive trap checks (a 48-h period with no captures). Thus, the total number of days over which trapping occurred varied among the releases, with the longest duration a 12-day recapture period. Collected samples were identified to species and sex. After identification, *Ae. aegypti* were examined under ultraviolet light to detect fluorescent dust, and marking status was recorded. The recapture rate was defined as the proportion of recaptured marked males in relation to the total number of marked males released for each collection day.

### Population parameters

#### Sterile male survival

Survival of released sterile males was estimated by the recapture rate per collection day with respect to the total number of marked sterile males released. The probability of daily survival (PDS) was estimated as the antilog of the regression slope of log(recaptured mosquitoes + 1), using date as a fixed parameter [23, 36]. The average life expectancy (ALE), defined as 1/− log_e_ (PDS), was used as our metric of longevity [23, 28, 37–39].

#### Sterile male dispersal

To generate estimates of dispersal for the SIT operational phase, three single-point MRRs (SP01, SP02, and SP03) were performed across the seasons. The minimum and maximum distances flown from the release point and positive trap location were defined using Vincenty’s formula for geodesic distance with an accurate ellipsoidal model of the earth [40]. For the mean distance traveled (MDT), the circular area was divided into nine concentric annuli, with a maximum radius of 450 m (Fig. 1c). The formula used to calculate MDT was MDT = ∑(ER × AD)/(total ER), where ER is the estimated recapture, and AD is the annulus distance. ER was estimated as the number of marked mosquitoes recaptured in each annulus divided by the number of traps in that annulus and multiplied by a factor used to correct the total number of traps. This correction factor was the area of the annulus divided by the total area in which the traps were set, multiplied by the total number of traps. AD was defined as the sum of the inner and outer annulus radius (from the release point) divided by 2 [41, 42]. The MDT value was used to extrapolate the distance estimates from our single-point releases to create a potential distribution (MDT-cloud) that allows one to visualize how far mosquitoes are likely to travel based on our MDT estimates, an estimated “MDT cloud.” By applying this approach to each multiple release point, the limits of the MDT-cloud were defined as the outermost line that the released mosquitoes were likely to have flown to. The flight range (FR) at 50% (FR_50_) and 90% (FR_90_) ER was determined as the antilog slope from the linear regression log_10_(AD + 1) ~ ER [37, 43].

#### Wild population size estimation

The Fisher-Ford index was used to estimate the wild population size for each MRR study performed for the first recapture day by considering the number of recaptured marked males and captured unmarked males [44]. The Fisher-Ford index was defined as (*S* × *n* × *M*/*m*) − *M*, where *n* is the number of captured unmarked (fertile) males, *M* is the total number of marked (sterile) males released, *m* is the number of recaptured marked (sterile) males, and* S* is the survival probability. The sterile:wild male ratio was determined by the number of marked recaptured males divided by the total number of captured unmarked wild males from the first day of trap capture after release.

### Statistical analysis

Statistical analyses were performed in R using RStudio environment [45, 46]. The outcome of the R script analyses is available as additional material (Additional file 1: Text S1). Relevant R packages and functions can also be found in the scripts provided in Additional file 1. Kernel density estimation interpolations (heat maps) were developed in QGIS version 3.16.9 Hannover [47], with a background map obtained from OpenStreetMap (Creative Commons Attribution-ShareAlike 2.0 license). Generalized linear models (GLMs) were used to evaluate male recapture parameters (recapture percentage or the number of recaptured males with distance, date, and MRR cycles as explanatory factors, using a binomial distribution for percentages and Poisson distribution for counts).

## Results

### Recapture success

Overall, we released 190,504 marked sterile male mosquitoes over our ~ 47-ha study area across the seven release cycles, with 3165 marked males recaptured, i.e. a mean recapture of ~ 1.5%. Table 2 summarizes each MRR study, presenting the number of marked sterile mosquitoes released, the recapture period [previously described as the number of days before a 2-day (48-h) period without recapture of marked males], the total number of marked (sterile) and unmarked (wild) male mosquitoes caught, and the male recapture rate. The proportion of sterile males recaptured varied from 1.73 to 4.10% for the single-point releases (average 2.70%), while in studies with multiple release points the proportion of sterile males recaptured ranged from 0.94 to 2.24% (average 1.39%). No significant difference was detected in the overall recapture proportions among the MRRs for the single-point releases (binomial GLM, deviance 0.462, *df* = 2, *P* = 0.638) or the multi-point releases (binomial GLM, deviance 1.371, *df* = 3, *P* = 0.269).

**Table 2:** Summary data of MRRs conducted across different time points in the selected study area of Captiva Island

MRR	Single points			Multiple points				Overall
	SP01	SP02	SP03	MP01	MP02	MP03	MP04	
Males reared (no.)	12,731	7399	2200	33,000	33,000	33,000	33,000	154,204
Males released (no.)	10,500	7273	2021	26,911	30,734	31,201	31,149	139,789
Mortality (%)	17.5	1.7	8.13	18.5	6.87	5.45	5.61	9.34
Marked recaptured males (no.)	239	126	83	473	187	294	703	2105
Recapture rate (%)	2.28	1.73	4.1	1.76	0.61	0.94	2.26	1.5
Trapping days	9	9	11	11	9	12	11	10.3
Unmarked males (no.)	150	60	769	8,044	1,436	15	769	11,243
Unmarked females (no.)	398	194	1214	7254	2235	220	1214	12,729
PDS	0.5	0.65	0.73	0.71	0.59	0.77	0.6	0.67
ALE (days)	1.45	2.29	3.24	2.92	1.87	3.91	1.95	2.46
Mosquito seasonality	Low ↓	Low ↑	Low ↓	High ↑	High ↓	Low ↓	Low ↓	-

Arrow direction indicates whether wild *Aedes aegypti* populations were expected to increase (upward-pointing arrow) or decrease (downward-pointing arrow) in the respective period

*PDS* Probability of daily survival,* ALE* average life expectancy; for other abbreviations, see Table 1

### Survival

**Fig. 2a, b:**
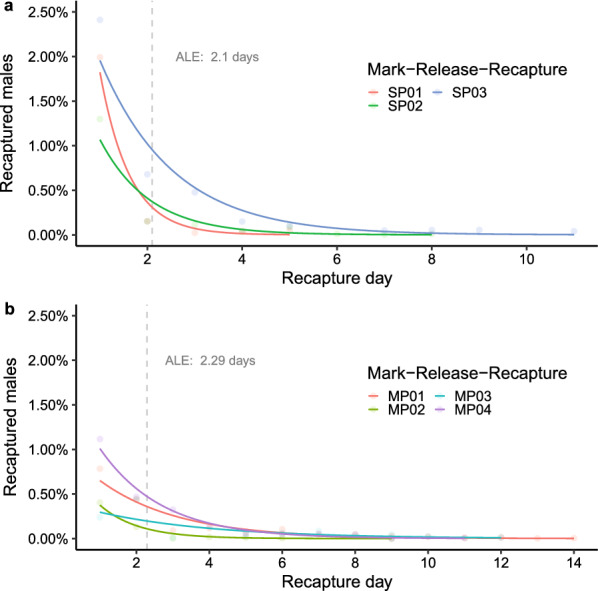
Daily mean proportion of marked males recaptured. Generalized linear model curves showing a binomial distribution of the recapture proportion of males as a the function of the day of recapture. **a** MRRs of single-point releases (SP01, SP02, SP03). **b** MRRs of multiple-point releases (MP01, MP02, MP03, and MP04). The dashed grey line represents the average life expectancy (*ALE*) for all the MRRs

Recapture rates were similar between single- and multiple-point releases (Fig. 2). The maximum time to recapture sterile males varied from 9 to 12 days among the studies, with the greatest number of recaptures occurring within the first 3 days after release (7.46% recapture/3 days) for both single- and multi-point releases. While the majority of marked, sterile males were recaptured within the first 3 days after release (Fig. 2), captures of the wild population fluctuated among days of trapping and across seasons. The longevity of released sterile males in the field, here estimated as ALE within each release, ranged from 1.45 in SP01 to 3.91 in MP03. The PDS ranged from 0.5 in SP01 to 0.77 in MP03 across all the releases in the study. Estimates of each parameter for every release are shown in Table 2.

### Dispersal from single-point releases

**Fig. 3a–c:**
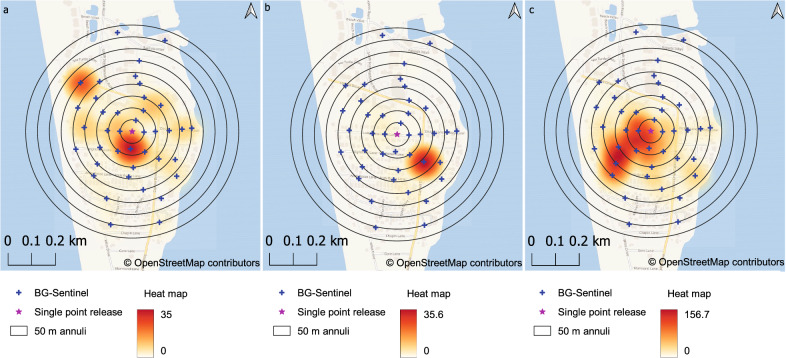
Spatial distribution of recaptured marked sterile males for each BG-Sentinel trap combined throughout the collection period. **a** SP01, **b** SP02, **c** SP03. This configuration was used to estimate dispersal parameters from a single-release point (the central pink star on each map). Annuli are 50 m from each other. Blue crosses represent the positions of the BG-Sentinel traps

We divided the circular area of our recapture zone into nine concentric annuli, each 50 m apart, from the most central point (Fig. 1), to calculate the MDT and FR. Across our three single-point releases (SP01, SP02, and SP03), we determined that sterile marked males had an MDT of 201.7 m, with a minimum DT and maximum DT of 36.2 and 404.5 m, respectively (Table 3; Fig. 3). The majority of sterile marked males recaptured were concentrated within the first 200-m radius from the release point (Fig. 3). The overall FR_50_ and FR_90_ estimates for sterile marked males were 132.97 and 188.4 m, respectively (Table 3).

**Table 3:** Dispersal estimates of released sterile male mosquitoes for three single-point MRRs on Captiva Island

MRR	Distance traveled (m)				
	MinDT	MaxDT	MDT	FR_50_	FR_90_
SP01	36.2	405.1	212.0	142.02	366.54
SP02	36.2	310.4	167.1	108.35	281.60
SP03	36.2	405.1	223.0	134.96	384.77
Overall	36.2	404.5	201.7	132.97	188.4

*MinDT* Minimum distance flown from the release point,* MaxDT* maximum distance flown from the release point, *MDT* mean distance traveled, *FR*_50_ flight range at 50% estimated recapture, *FR*_50_ flight range at 90% estimated recapture

There was a marginally significant difference in marked sterile male dispersal (AD) among the three single-point releases (Poisson GLM, deviance − 2.055, *df* = 2, *P* = 0.04), but pairwise differences in dispersal distance among the three single-point releases were not statistically significant. Similarly, while there was a statistically significant difference in the proportion of sterile males recaptured per trap among releases (binomial GLM, deviance − 28.6, *df* = 2, *P* < 0.001), there were no significant pairwise differences among the three single-point releases (log odds ratio of recapture probability among the traps, SP01 vs SP02, SP01 vs SP03, and SP02 vs SP03; for further details, see Additional file 2: Fig. S1).

### Dispersal from multiple-point releases

**Fig. 4:**
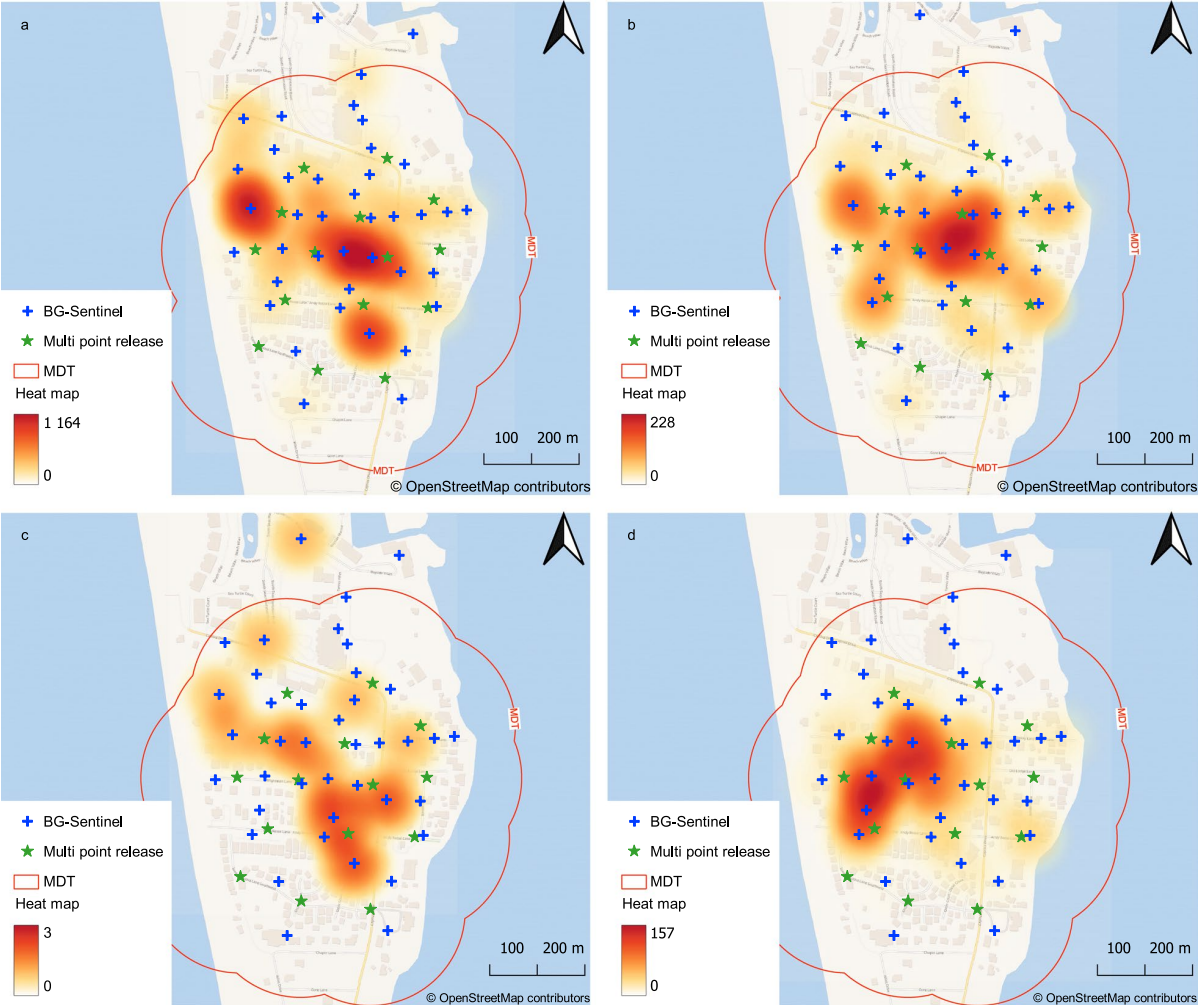
Spatial distribution of recaptured marked sterile males showing clusters within the release area observed in the multiple-point MRR studies (**a** MP01, **b** MP02, **c** MP03, **d** MP04). The MDT was based on the cumulative estimated recapture, the trap density in the annuli area, and male capture from the single-point releases. The heat maps represent kernel density estimations, which took into consideration the BG-Sentinel capture radius of 50 m and the number of mosquitoes captured. Blue crosses represent BG-Sentinel positions, while green stars indicate release points

The MDT data from SP01, SP02, and SP03 were extrapolated to represent a potential dispersal cloud when taking into consideration the 14 additional release points of each MRR-multi-point release (MP01, MP02, MP03, and MP04). For this calculation, the MDT was used as a reference distance for each release point to give a final release perimeter of potential flight around the release points. This perimeter was defined as the MDT-cloud. Figure 4 shows each MRR-multi-point release, with the marked male distribution within the MDT-cloud. The multiple-point releases showed that, among our studies (according to the time of year), the most central area had the greatest recapture frequencies. However, recapture frequencies changed among traps during the MRR-multi-point releases, which resulted in captures outside the predicted MDT-cloud (Fig. 4C).

### Estimates of wild population size

Population size estimates for wild *Ae. aegypti* fluctuated through time as conditions in the field changed from favorable to hostile for mosquito growth and activity, which reflected periods of population increase and decline, respectively (Table 4). The first multiple-point release (MP01) was conducted in May during the time that wild *Ae. aegypti* activity was ramping up toward that of the typical high population period on Captiva, which occurs in July, and the estimated population size recorded using the Fisher-Ford index was ~ 13,000 wild (fertile) males in our 47-ha MRR area. The second release (MP02) took place in August, not long after the peak of seasonal activity, as populations were still high but beginning to decline (~ 27,000 wild males estimated via the Fisher-Ford index). MP03 took place in December, the low-density season (~ 653 wild males estimated via the Fisher-Ford index), and finally, MP04 took place in April, a time during which the wild *Ae. aegypti* population had begun to increase rapidly again (~ 6000 males estimated via the Fisher-Ford index). From MP01 to MP02 (12 weeks apart, May–August), we observed that the population more than doubled, followed by a drastic reduction of ~ 98.2% from MP02 to MP03 (16 weeks apart, August–December). The overall sterile:wild male ratio induced by our releases was low due to the wild population sizes; for MP01, the ratio was 0.059 in May; for MP02, the ratio was 0.16 in August; and for MP04, the ratio was 0.98 in April; meanwhile for MP03 the ratio was 21 in December when the wild population was at its lowest (Table 4).

**Table 4:** Male *Aedes aegypti* population estimation from the MRR studies performed in 2019 and 2020 on Captiva Island

MRR	Mosquito seasonality	Fisher-Ford index	Sterile:wild ratio
MP01	High ↑	13167.6	0.059
MP02	High ↓	26857.9	0.160
MP03	Low ↓	652.8	21
MP04	Low ↑	6055.8	0.98
Average		11683.5	5.55

Arrow direction indicates increasing (upward-pointing arrow) or decreasing (downward-pointing arrow) wild *Ae. aegypti* populations in the respective period

## Discussion

Effective mosquito control using SIT requires the collection of baseline data on local wild population dynamics which makes it possible to efficiently plan the suppression phase for that specific location, and includes population size estimates and seasonal patterns of abundance, as these will dictate when sterile male releases should occur [18]. In addition to estimates of local pest population sizes and dynamics, knowledge of sterile male dispersal and survival in the field is used to determine the numbers of sterile males that must be released to suppress the local population, as well as the frequency of releases. These data can be obtained through sequential MRR studies conducted across the seasons to track the local pest mosquito population in the target area [48–50]. While the marking methodologies differed across the MRR studies presented here, the goals of these studies were not to compare different marking techniques but rather to best estimate wild *Ae. aegypti* population size, along with sterile male dispersal, FR, and longevity. Alterations in the marking protocols reflect operational improvements that were determined through experience accumulated when marking sterile males for release.

The results presented here provide essential information for SIT planning and implementation for *Ae. aegypti* on Captiva Island. Typically, the aim of SIT programs is to release enough sterile males to overflood the population of wild males during low wild population density, to quickly reach ratios of 10–100 sterile males for each wild male, thereby reducing opportunities for wild females to mate with fertile wild males [49, 50]. Thus, it is important to estimate the wild male mosquito abundance to plan how many sterile males must be reared and released to affect the wild population. The ratio of marked sterile males to unmarked wild males taken from consecutive MRR studies provides a robust general idea about the release densities of sterile males required based on the seasonal dynamics [49, 50]. Some studies suggest that Fisher-Ford index estimations may overestimate wild populations due to a lack of knowledge about some population parameters, such as birth rate, mortality, age structure, immigration and emigration, and competition, etc. [51–53].

Additionally, population modeling is demanding due to the number of parameters needed. For effective SIT, the level of accuracy of population estimation at the initial stages is critical and quite challenging [51, 54]. However, practitioners can also use the sterile:wild (fertile) ratio as a viable alternative to population estimation to estimate the number of sterile males needed for release in a site to achieve a minimum sterile:wild male ratio of 10:1, which have been shown to generally cause wild population reduction. However, if production of sterile males allows overflooding ratios that are even greater than 10:1, the SIT may be even more effective; indeed, in some programs, as many as 100 sterile males are released for each wild male [51]. In our study there were, on average, 3.64 sterile males for each wild (fertile) one, which indicates that sterile male production needs to be increased in our operational releases, i.e. to reach the numbers necessary to have at least 10 sterile males for each wild male. Furthermore, releases for population suppression should ideally start early in the season when wild populations naturally have lower abundances so that the initial overflooding ratios are very high, which in our case is possible given the production capacity at our facility [17, 49]. Our current results form a basis for planning and implementing releases. We will also implement consistent monitoring of wild females with periodic releases of marked males to evaluate sterile:wild male ratios while the suppression phase is implemented, allowing future adjustments to the numbers of sterile males released to achieve successful suppression [55, 56].

Previous trapping studies on Captiva Island have shown substantial seasonal variation in *Ae. aegypti* adult densities (R. Morreale, unpublished data), with populations low over the cooler dry season months (October through April) and population expansion during the warmer, rainy season months (May through September). As expected, our estimates of wild male population size fluctuated across the MRR studies, from relatively low in December (~ 650 in the study area for MP03) to growing in April (~ 6000 in the study area for MP04) to very high in August (~ 26,800 in the study area for MP02) [50, 57, 58]. For the most effective population suppression over this ~ 47-ha urban site, we should aim to release ~ 500,000 sterile males per week at the beginning of spring before wild populations build up, to prevent early population growth.

The number of males needed to drive successful population control is based on the size of the wild population and the performance of the released sterile males in the field [18, 51]. The daily male recapture rate, PDS, and ALE are fundamental parameters for assessing the quality of the males reared and released. These parameters are helpful for deciding how often sterile males should be released in an operational program. Despite the fact that our MRR insects were submitted to sterilization through irradiation, our results were close to those found by Maciel-de-Freitas et al. [59] in a Brazilian study, in which PDS varied from 0.7 to 0.9, while in our study PDS was 0.67. The large range in PDS in the former study was reflected in the range in ALE, from 3 to 12 days, while in our study ALE was 2.46 days (or approximately 3 days). This supports the need to release high-quality males into the field to increase the chances of wild females mating with sterile males [60–62]. This ALE, which we consider a minimum, agrees with that of similar, established SIT pilot trials (not only for mosquitoes), and indicates that the release of sterile males, especially in our area, should occur two times per week with 2–3 days between releases. For example, the success of a program used to suppress the codling moth (*Cydia pomonella*), which employed a release frequency of two times per week, is considered an excellent example of successful SIT application [63]. The interval is of fundamental importance since the age of males can also impact their recapture rate, as described by Harrington et al. [64], who found that recapture rate decreases as mosquitoes age, with a small difference during dry and wet seasons, with a recaptured proportion of less than 5% in both periods.

Information on dispersal and distribution are also fundamental to determining release patterns of sterile males for population suppression programs, to ensure that releases occur where needed and to minimize releases in sites where they are not as important for mosquito control [18, 28]. Furthermore, dispersal studies may identify barriers specific to a particular location that may affect sterile male distribution or the migration of wild insects into the study area. The MDT of 195 m reported here is comparable to the long MDT, 240 m, reported in 2019 from another American study, for non-irradiated *Ae. aegypti* males [65], despite the differences between our site, on a Florida barrier island, and the former’s, which was in central California. Specifically, southwest Florida has a humid subtropical climate, while the study in California took place in an area with a cold semi-arid climate, yet male *Ae*. *aegypti* appeared to disperse over roughly the same distance at both locations [66]. Another study from the US reported similar results, with an overall MDT of 241.82 m and a maximum of 428.45 m for non-irradiated insects [67]. This indicates that, despite the fact our males were irradiated, they were fit enough to perform similarly to non-irradiated insects. The FR_50_ of 133.5 m reported here suggests that, for population suppression, we should develop a release plan based on a distance of ~ 100 m between release points to ensure even coverage of sterile males throughout our population suppression site. While a maximum radius of 133 m between release points could be considered, we feel that having a release radius of ~ 100 m will provide an excellent overlap of released mosquitoes to improve the probability of sterile male mating success; this distance is also close to the average MDT, 105.69, estimated by Moore and Brown [68] from 27 experiments.

As Captiva is an offshore island, the population of mosquitoes there is considered mostly closed, which increases the potential for successful suppression, and even eradication, of *Ae. aegypti* from the island. Our aim of releasing ~ 500,000 sterile males per week in our ~ 47-ha focal area in the most densely populated northern part of the island may need to be revised if immigration of fertile *Ae. aegypti* from the contiguous southern end of the island into our initial suppression area occurs. Ultimately, our goal is to eliminate *Ae. aegypti* across the entire ~ 240-ha island, but this will require substantially increasing the production of sterile males to cover the additional area.

## Conclusions

Knowledge of the spatial distribution of trap captures yields essential information about a mosquito population in real time, and helps us identify areas of interest and hot spots that may need additional efforts or higher numbers of sterile males released [61, 68]. We used single- and multiple-release points across our studies to take advantage of the unique type of information acquired using these different approaches. We also showed that it is possible to perform single- and multi-point releases simultaneously, which helps to maximize the impact of sterile male releases on population density and dispersal. Finally, we argue that collecting data from the field through a baseline data collection system in parallel with sequential MRR studies is fundamental to the planning of an operational SIT phase. The information gained through both efforts generates a solid and stable basis from which to guide release and monitoring measures to achieve mosquito population suppression on Captiva Island.

## Supplementary Information

**Additional file 1: Text S1.** Statistical analysis and R code.

**Additional file 2: Figure S1.** BG-Sentinel male collections (marked and unmarked) across our seven MRR studies including single-point releases (**a** SP01, SP02, SP03) and multiple-point releases (**b** MP01, MP02, MP03, MP04) covering seasonal periods of relatively low and high mosquito densities across 2019 and 2020 on Captiva Island.

## Data Availability

The datasets used and analyzed during the current study are available from the corresponding author on reasonable request.
