# Supplementary material for: A sterile insect technique pilot trial on Captiva Island: defining mosquito population parameters for sterile male releases using mark–release–recapture
Source: Parasit Vectors. 2022 Nov 1;15:402. doi: 10.1186/s13071-022-05512-3 (PMC9628054; doi:10.1186/s13071-022-05512-3)
Supplement: Supplementary file 1 — Additional file 1: Text S1. Statistical analysis and R code. [file 13071_2022_5512_MOESM1_ESM.pdf]

Additional file 1: Text S1

Statistical Analysis and R code

2022-03-15

```
library(tidyverse)

## -- Attaching packages --------------------------------------- tidyverse
1.3.1 --

## v ggplot2 3.3.5     v purrr   0.3.4
## v tibble  3.1.6     v dplyr   1.0.8
## v tidyr   1.2.0     v stringr 1.4.0
## v readr   2.1.2     v forcats 0.5.1

## -- Conflicts ------------------------------------------
tidyverse_conflicts() --
## x dplyr::filter() masks stats::filter()
## x dplyr::lag()    masks stats::lag()

library(knitr) # kable()
library(ggpubr) #stat_compare_means ggscatter
library(geosphere) #calculate de distance between the release point and trap
library(glmm)

## Loading required package: trust

## Loading required package: mvtnorm

## Loading required package: Matrix

##
## Attaching package: 'Matrix'

## The following objects are masked from 'package:tidyr':
##
##     expand, pack, unpack

## Loading required package: parallel

## Loading required package: doParallel

## Loading required package: foreach

##
## Attaching package: 'foreach'

## The following objects are masked from 'package:purrr':
##
##     accumulate, when
```

```
## Loading required package: iterators

library(lubridate)

##
## Attaching package: 'lubridate'

## The following objects are masked from 'package:base':
##
##     date, intersect, setdiff, union

# Organizing data bank #
data.bank.mrr2<-function(data){
  a<-data%>%filter(gender!="female",
marking_status=="unmarked")%>%group_by(date1)%>%
    summarise(unmarked=sum(n_adults))
  b<-data%>%filter(gender!="female",
marking_status=="marked")%>%group_by(date1)%>%
    summarise(marked=sum(n_adults))
  mrr_data<-full_join(a,b,by="date1")
  mrr_data[is.na(mrr_data)]<-0
  mrr_data$captured<-mrr_data$unmarked+mrr_data$marked
  mrr_data
}

# PDS&ALE #
pds.ale<-function(data){
  model<-lm(log10(marked+1)~date1, data=data)
  PDS<-as.numeric(10^coef(model)[2])
  ALE<-1/-log(PDS)
  result<-data.frame(PDS=round(PDS, 2), ALE=round(ALE,2))
  result
}

# Number released #
total.released<-function(data){
  result<-data%>%group_by(round)%>%
    summarise(total_dead=sum(no.pupae,no.adult),
              estimated=sum(estimates)/length(unique(evaluation)),
              survival=1-(total_dead/estimated),
              released=estimated*survival)
  result
}

# Formulas for Distance #
annulus.area<-function(R,r){
  for(value in r)
    if (value == 0) {
      A<-pi*R^2
    }else{
```

```
      A<-pi*(R^2-r^2)
    }
  data.frame(annulus=R, area=round(A,1))
}

model.equation <- function(model, ...) {
  format_args <- list(...)

  model_coeff <- model$coefficients
  format_args$x <- abs(model$coefficients)
  model_coeff_sign <- sign(model_coeff)
  model_coeff_prefix <- case_when(model_coeff_sign == -1 ~ " - ",
                                  model_coeff_sign == 1 ~ " + ",
                                  model_coeff_sign == 0 ~ " + ")
  model_eqn <- paste(strsplit(as.character(model$call$formula), "~")[[2]], #
'y'
                     "=",
                     paste(if_else(model_coeff[1]<0, "- ", ""),
                           do.call(format, format_args)[1],
                           paste(model_coeff_prefix[-1],
                                 do.call(format, format_args)[-1],
                                 " * ",
                                 names(model_coeff[-1]),
                                 sep = "", collapse = ""),
                           sep = ""))
  return(model_eqn)
}

MDT<-function(data){
  er_sum<-sum(data$ER)
  er_dist<-data$ER*data$a_distance
  mdt<-round(sum(er_dist)/er_sum,1)
  mdt
}

correction.factor<-function(area,total_area,total_trap){
  a<-(area$area/total_area)*total_trap
  data.frame(annulus=area$annulus,CF=a)
}

flight.range<-function(data, tt="BG Sentinel", c="pink"){
  d_round<-data$round
  d_annulus<-data$annulus
  d_a_distance<-data$a_distance
  d_ER<-data$ER
  d_cumsum<-cumsum(data$ER)

  flight_range<-data.frame(round=d_round, annulus=d_annulus,
a_distance=d_a_distance,
```

```
                                  ER=d_ER, cumsum=d_cumsum)

  flight_model<-lm(log10(a_distance+1)~cumsum, data = flight_range)
  intercept<-flight_model$coefficients[[1]]
  slope<-flight_model$coefficients[[2]]

  fr90<-(10^(intercept+slope*(max(flight_range$cumsum)*0.9)))-1
  fr50<-(10^(intercept+slope*(max(flight_range$cumsum)*0.5)))-1
  fr<-c((intercept+slope*(max(flight_range$cumsum)*0.5)),
        (intercept+slope*(max(flight_range$cumsum)*0.9)))
  plot1<-ggplot(data = flight_range, aes(x=cumsum, y=log10(a_distance+1)))+
    geom_point()+geom_smooth(method = "lm")+
    geom_hline(yintercept = log10(c(fr50, fr90)+1))
  return(list(fr50,fr90,plot1))
}

#### unique MRR features ####
release_point<-data.frame(long=-82.19055556, lat=26.52370278)
release_multipoints<-read.csv("./multipoints_lat_long_table.csv")
trap_position<-read.csv("./mrr_trap_location.csv")

#### MRR collection data ####
mrr_data<-read.csv("./MRR_all_collection.csv")
mrr_data$date<-dmy(mrr_data$date)
mrr_data$f_week_date<-epiweek(mrr_data$date)
mrr_data$f_year_date<-year(mrr_data$date)

mrr_data$release<-dmy(mrr_data$release)
mrr_data$f_week_release<-epiweek(mrr_data$release)
mrr_data$f_year_release<-year(mrr_data$release)

mrr_data$date1<-mrr_data$date-mrr_data$release
mrr_data$date2<-as.numeric(mrr_data$date1)

mrr_data2<-left_join(mrr_data, trap_position, by="trap_ID")
write.csv(mrr_data2,"MRR_all_collection2.csv")

# Calculating the PDS for graphic purpose #
PDS_total<-pds.ale(data.bank.mrr2(mrr_data))

PDS_singles<-
pds.ale(data.bank.mrr2(mrr_data%>%filter(round%in%c("SP01","SP02","SP03"))))

PDS_multiples<-
pds.ale(data.bank.mrr2(mrr_data%>%filter(!round%in%c("SP01","SP02","SP03"))))

#### MRR release data ####
mrr_release<-read.csv("./MRR_all_release.csv")
mrr_release$date<-as.Date(mrr_release$date, format='%Y-%m-%d')
```

```
mrr_release_summary<-total.released(mrr_release)

#### MRR distance data ####
mrr_distance<-mrr_data%>%filter(round%in%c("SP01","SP02","SP03"))
mrr_distance<-left_join(mrr_distance, mrr_release_summary, by="round")

mrr_distance$distance<-distVincentyEllipsoid(release_point,
                                             cbind(mrr_distance$long,
mrr_distance$lat))
mrr_distance$recap_rate<-mrr_distance$n_adults/mrr_distance$released

model_distance_date<-glmm(n_adults~0+distance+date,
           random = (~0+round), varcomps.names = "MRR",
           m = 10^2, data = mrr_distance%>%
             filter(gender=="male", marking_status=="marked"),
           family.glmm = poisson.glmm)
summary(model_distance_date)

##
## Call:
## glmm(fixed = n_adults ~ 0 + distance + date, random = (~0 + round),
##     varcomps.names = "MRR", data = mrr_distance %>% filter(gender ==
##         "male", marking_status == "marked"), family.glmm = poisson.glmm,
##     m = 10^2)
##
##
## Link is: "log"
##
## Fixed Effects:
##          Estimate Std. Error z value Pr(>|z|)
## distance        0          0  -8.636   <2e-16 ***
## date            0          0  16.332   <2e-16 ***
## ---
## Signif. codes:  0 '***' 0.001 '**' 0.01 '*' 0.05 '.' 0.1 ' ' 1
##
##
## Variance Components for Random Effects (P-values are one-tailed):
##     Estimate Std. Error z value Pr(>|z|)/2
## MRR    0.152      0.131    1.16      0.123

##### MRR Distance Table #####
mrr_distance%>%
  filter(round%in%c("SP01","SP02","SP03"),gender=="male",
marking_status=="marked")%>%
  group_by(round)%>%
  summarise(sum_adults=sum(n_adults),
            mean_dist=mean(distance),
            median_dist=median(distance),
            min_dist=min(distance),
            max_dist=max(distance))
```

```
## # A tibble: 3 x 6
##   round sum_adults mean_dist median_dist min_dist max_dist
##   <chr>      <int>     <dbl>       <dbl>    <dbl>    <dbl>
## 1 SP01         239      152.        143.      36.2     405.
## 2 SP02         126      112.         94.8     36.2     310.
## 3 SP03          83      120.         94.7     36.2     405.

mrr_distance<-mrr_distance%>%separate(trap_ID,c("trap","ID"),"_")

distance_data<-mrr_distance%>%filter(marking_status=="marked")%>%
  group_by(trap, round)%>%summarise(males=sum(n_adults))

## `summarise()` has grouped output by 'trap'. You can override using the
## `.groups` argument.

annulus_outer<-seq(50,450,50)
annulus_inner<-seq(00,400,50)
total_area<-pi*max(annulus_outer)^2

mrr_distance<-mrr_distance%>%mutate(annulus = case_when(
  between(distance, 0, 50) ~ 50,
  between(distance, 51, 100) ~ 100,
  between(distance, 101, 150) ~ 150,
  between(distance, 151, 200) ~ 200,
  between(distance, 201, 250) ~ 250,
  between(distance, 251, 300) ~ 300,
  between(distance, 301, 350) ~ 350,
  between(distance, 351, 400) ~ 400,
  between(distance, 401, 450) ~ 450))

mrr_annulus<-mrr_distance%>%
  filter(gender=="male", marking_status=="marked")%>%
  group_by(round, annulus)%>%
  summarise(sum_trap=length(unique(n_adults)),
            recapture=sum(n_adults))

## `summarise()` has grouped output by 'round'. You can override using the
## `.groups` argument.

mrr_distance%>%
  filter(gender=="male", marking_status=="marked")%>%
  #group_by(round)%>%
  summarise(max=max(distance),min=min(distance))

##        max      min
## 1 404.5268 36.19608

annulus_distance<-data.frame(annulus=annulus_outer,
                             a_distance=(annulus_inner+annulus_outer)/2)
annulus_area<-annulus.area(annulus_outer,annulus_inner)
```

```
CF<-correction.factor(annulus_area, total_area, 40)

mrr_annulus<-left_join(mrr_annulus, CF, by="annulus")
mrr_annulus<-left_join(mrr_annulus, annulus_distance, by="annulus")
mrr_annulus<-mrr_annulus%>%mutate(ER=(recapture/sum_trap)*CF)

MDT_table<-sapply(split(mrr_annulus, mrr_annulus$round), FUN=MDT)
t_MDT_table<-as.data.frame(MDT_table)
MDT_table<-data.frame(type_trap=rownames(t_MDT_table),
MDT=t_MDT_table$MDT_table)
MDT_table

##   type_trap   MDT
## 1      SP01 212.0
## 2      SP02 167.1
## 3      SP03 223.0

MDT(mrr_annulus)

## [1] 201.7

FR_SP01<-flight.range(mrr_annulus%>%filter(round=="SP01"))
FR_SP02<-flight.range(mrr_annulus%>%filter(round=="SP02"))
FR_SP03<-flight.range(mrr_annulus%>%filter(round=="SP03"))

MRR_FR<-flight.range(mrr_annulus)

#### Recapture rate ####

#### Recapture Summary ####
recap_data<-mrr_data%>%
  filter(gender=="male", round%in%c("SP01", "SP02","SP03", "MP01", "MP02",
"MP03", "MP04"),
         marking_status=="marked")%>%
  group_by(f_year_date,f_week_date,round,
date1)%>%summarise(total_recapt=sum(n_adults))

## `summarise()` has grouped output by 'f_year_date', 'f_week_date', 'round'.
You
## can override using the `.groups` argument.

recap_data<-merge(x = recap_data,y = mrr_release_summary[,c(1,5)], by =
"round",all.x = TRUE)
recap_data$recap_rate<-recap_data$total_recapt/recap_data$released

glm_data<-mrr_data%>%
  filter(marking_status=="marked", gender=="male", round%in%c("MP01", "MP02",
"MP03", "MP04"))
summary(aov(glm(n_adults~round*date1, data = glm_data)))
```

```
##              Df Sum Sq Mean Sq F value   Pr(>F)
## round         3    733   244.3   15.59 1.03e-09 ***
## date1         1   1203  1202.8   76.78  < 2e-16 ***
## round:date1   3    634   211.4   13.49 1.75e-08 ***
## Residuals   506   7927    15.7
## ---
## Signif. codes:  0 '***' 0.001 '**' 0.01 '*' 0.05 '.' 0.1 ' ' 1
```

```
model_n_adult_date1_round<-glmm(n_adults~0+date1,
            random = (~0+round), varcomps.names = "MRR",
            m = 10^2, data = glm_data,
            family.glmm = poisson.glmm)

summary(model_n_adult_date1_round)
```

```
##
## Call:
## glmm(fixed = n_adults ~ 0 + date1, random = (~0 + round), varcomps.names =
"MRR",
##     data = glm_data, family.glmm = poisson.glmm, m = 10^2)
##
##
## Link is: "log"
##
## Fixed Effects:
##       Estimate Std. Error z value Pr(>|z|)
## date1        0          0  -20.13   <2e-16 ***
## ---
## Signif. codes:  0 '***' 0.001 '**' 0.01 '*' 0.05 '.' 0.1 ' ' 1
##
##
## Variance Components for Random Effects (P-values are one-tailed):
##     Estimate Std. Error z value Pr(>|z|)/2
## MRR        0          0   1.411     0.0791 .
## ---
## Signif. codes:  0 '***' 0.001 '**' 0.01 '*' 0.05 '.' 0.1 ' ' 1
```

```
model_n_adult_date1_trap<-glmm(n_adults~0+date1,
            random = (~0+trap_ID), varcomps.names = "Trap ID",
            m = 10^2, data = glm_data,
            family.glmm = poisson.glmm)

summary(model_n_adult_date1_trap)
```

```
##
## Call:
## glmm(fixed = n_adults ~ 0 + date1, random = (~0 + trap_ID), varcomps.names
= "Trap ID",
##     data = glm_data, family.glmm = poisson.glmm, m = 10^2)
##
##
```

```
## Link is: "log"
##
## Fixed Effects:
##       Estimate Std. Error z value Pr(>|z|)
## date1        0          0  -25.12   <2e-16 ***
## ---
## Signif. codes:  0 '***' 0.001 '**' 0.01 '*' 0.05 '.' 0.1 ' ' 1
##
##
## Variance Components for Random Effects (P-values are one-tailed):
##         Estimate Std. Error z value Pr(>|z|)/2
## Trap ID        0          0   4.443   4.43e-06 ***
## ---
## Signif. codes:  0 '***' 0.001 '**' 0.01 '*' 0.05 '.' 0.1 ' ' 1
```

```
#### Distance vs Recapture ####
model_recapture<-glmm(as.integer(distance)~0+recap_rate+date2,
             random = (~0+round), varcomps.names = "MRR",
             m = 10^3, data = mrr_distance%>%filter(gender=="male",
marking_status=="marked"),
             family.glmm = poisson.glmm)
summary(model_recapture)
```

```
##
## Call:
## glmm(fixed = as.integer(distance) ~ 0 + recap_rate + date2, random = (~0 +
##     round), varcomps.names = "MRR", data = mrr_distance %>% filter(gender
==
##     "male", marking_status == "marked"), family.glmm = poisson.glmm,
##     m = 10^3)
##
##
## Link is: "log"
##
## Fixed Effects:
##            Estimate Std. Error z value Pr(>|z|)
## recap_rate     -440         20  -28.59   <2e-16 ***
## date2             0          0  -15.41   <2e-16 ***
## ---
## Signif. codes:  0 '***' 0.001 '**' 0.01 '*' 0.05 '.' 0.1 ' ' 1
##
##
## Variance Components for Random Effects (P-values are one-tailed):
##     Estimate Std. Error z value Pr(>|z|)/2
## MRR       30         20   1.225       0.11
```

```
model_distance<-glmm(recap_rate~0+distance,
             random = (~0+round), varcomps.names = "MRR",
             m = 10^3, data = mrr_distance%>%filter(gender=="male",
marking_status=="marked"),
```

```
              family.glmm = binomial.glmm)
summary(model_distance)

##
## Call:
## glmm(fixed = recap_rate ~ 0 + distance, random = (~0 + round),
##     varcomps.names = "MRR", data = mrr_distance %>% filter(gender ==
##         "male", marking_status == "marked"), family.glmm = binomial.glmm,
##     m = 10^3)
##
##
## Link is: "logit (log odds)"
##
## Fixed Effects:
##          Estimate Std. Error z value Pr(>|z|)
## distance        0          0  -2.055   0.0399 *
## ---
## Signif. codes:  0 '***' 0.001 '**' 0.01 '*' 0.05 '.' 0.1 ' ' 1
##
##
## Variance Components for Random Effects (P-values are one-tailed):
##     Estimate Std. Error z value Pr(>|z|)/2
## MRR        0          0   0.278       0.39
```
