# Supplementary material for: A sterile insect technique pilot trial on Captiva Island: defining mosquito population parameters for sterile male releases using mark–release–recapture
Source: Parasit Vectors. 2022 Nov 1;15:402. doi: 10.1186/s13071-022-05512-3 (PMC9628054; doi:10.1186/s13071-022-05512-3)

Additional Material

Additional file 2. BG-Sentinel male collections (marked and unmarked) across our seven MRR studies including single-point releases (A - SP01, SP02, SP03) and multiple-point releases (B - MP01, MP02, MP03, MP04) covering seasonal periods of relatively low and high mosquito densities across 2019-2020 on Captiva Island, FL, United States.

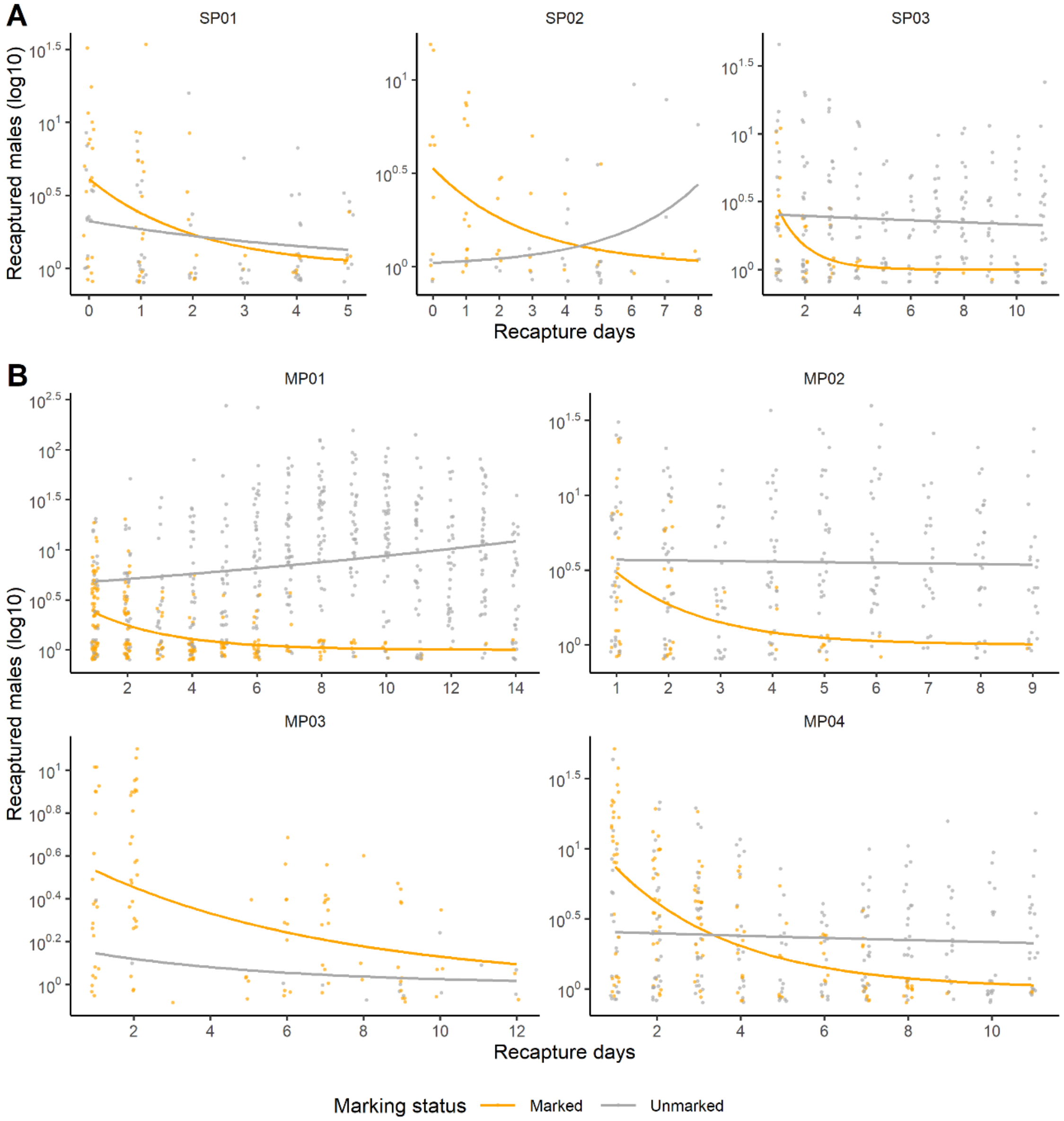

Supplement: Supplementary file 2 — Additional file 2: Figure S1. BG-Sentinel male collections (marked and unmarked) across our seven MRR studies including single-point releases (a SP01, SP02, SP03) and multiple-point releases (b MP01, MP02, MP03, MP04) covering seasonal periods of relatively low and high mosquito densities across 2019 and 2020 on Captiva Island. [file 13071_2022_5512_MOESM2_ESM.pdf]
